# More Than One-Third of Pregnant Women in Ethiopia Had Dropped Out From Their ANC Follow-Up: Evidence From the 2019 Ethiopia Mini Demographic and Health Survey

**DOI:** 10.3389/fgwh.2022.893322

**Published:** 2022-07-14

**Authors:** Mandaras Tariku, Biruk Shalmeno Tusa, Adisu Birhanu Weldesenbet, Nebiyu Bahiru, Daniel Berhanie Enyew

**Affiliations:** ^1^Department of Psychiatry, College of Health and Medical Sciences, Haramaya University, Haramaya, Ethiopia; ^2^Department of Epidemiology and Biostatistics, School of Public Health, College of Health and Medical Sciences, Haramaya University, Haramaya, Ethiopia; ^3^Department of Public Health and Health Policy, School of Public Health, College of Health and Medical Sciences, Haramaya University, Haramaya, Ethiopia

**Keywords:** ANC dropout, pregnant women, Ethiopia, Demographic and Health Survey data, DHS

## Abstract

**Background:**

In Ethiopia, the magnitude of antenatal care (ANC) practice and institutional delivery is low as compared with developed countries. The majority of the pregnant women have not completed their ANC follow-up and only 43% of women have reached the four and above ANC. This study was conducted to determine the magnitude of ANC dropout and associated factors among pregnant women in Ethiopia.

**Methods:**

Secondary data analysis was conducted using the 2019 Ethiopia Mini Demographic and Health Survey 2019 (2019 EMDHS). The sample was selected using a stratified, two-stage cluster sampling design and the data were analyzed using the binary logistic regression model to identify factors associated with ANC dropout. Adjusted odds ratio (AOR) with 95% CI was reported to declare significance and strength of association. A total weighted sample of 2,143 women who had antenatal care follow-up during pregnancy was included. In the multivariate logistic regression analysis, variables having a *p*-value < 0.05 were considered to have a significant association with ANC dropout.

**Result:**

The magnitude of ANC dropout was 39.12% (95% CI: 37.07 and 41.20%) among women who had ANC follow-up in Ethiopia. Aged 30–49 years [AOR = 0.71; 95% CI: (0.54, 0.94)], attended primary [AOR = 0.79; 95% CI: (0.62, 0.99)], secondary [AOR = 0.63; 95% CI: (0.44, 0.87)], and higher education [AOR = 0.39; 95% CI: (0.25, 0.62)], were in first trimesters [AOR = 0.49; 95% CI: (0.40, 0.60)] at the time of first ANC visit, and had access to laboratory service [AOR = 0.25; 95% CI: (0.13, 0.51)] were found to be a negative significant associated factors of ANC dropouts, whereas being rural resident [AOR = 1.53; 95% CI: (1.11, 2.10)] has a positive significant association with ANC dropouts.

**Conclusion:**

More than one-third of the pregnant women in Ethiopia had dropped out from their ANC follow-up in the study period. Being old-aged, educated, urban resident, having a first ANC visit in the first trimester, and having access to laboratory service were negatively associated with ANC dropouts. Therefore, we recommended encouraging women to have ANC visit at an early stage of pregnancy and conducting basic laboratory investigations during their visit. When undertaking that, due attention should be given to young, uneducated, and rural dweller women.

## Background

Antenatal care (ANC) is the care provided by skilled healthcare professionals to pregnant women and adolescent girls in order to ensure the best health conditions for both the mother and baby during pregnancy (1). Since the introduction of focused antenatal care in 2002 by the WHO, there is a stunning change in the utilization of ANC follow-up in low- and middle-income countries and it was then recommended at least four ANC visits for pregnant women (2–4). ANC is one of the strategies designed to reduce neonatal mortality and maintain maternal health by teaching women to have more access to maternal health services and get ready for their newborns (5).

In low-income countries, the number of women attending antenatal care has gradually increased from 64% in 1990 to about 81% in 2009. However, only 39% of pregnant women attended four times or more antenatal care during 2000–2010. Maternal mortality was estimated at 216 globally and almost all (95%) happened in developing countries (6) and the rate was 412 in Ethiopia (7). Although the country has registered the dramatic change in reducing maternal and child mortality, the health of pregnant women and their children has remained a public health problem (8).

The health authority and governmental and nongovernmental organizations are paying attention and working toward reducing maternal and child mortality by providing a roadmap for ending preventable deaths of women, children, and adolescents by 2030 and helping them achieve their potential for and rights to health and wellbeing in all the settings (9). These strategies have three main objectives: ending preventable deaths, ensuring health and wellbeing, and expanding enabling environments, which were aligned with 17 targets within nine of the Sustainable Development Goals (10).

In Ethiopia, the magnitude of antenatal care practice and institutional delivery was low as compared with the ones in developed countries. The majority of the pregnant women have not completed their ANC follow-up and only 43% of women have reached the four and above ANC. Around 48% of the pregnant women had institutional delivery (11). Ongoing rates of dropout from their ANC follow-up have a negative impact on the pregnant women's health status and take the lion's share of high morbidity and mortality (11, 12).

The ANC dropout rate was more common in developing countries, particularly in sub-Saharan African countries where the ANC dropout rate was high (13, 14). Different scholars thought that various factors hindering the pregnant women make ANC dropout rate. Several studies suggested that socioeconomic factors such as poverty, accessibility of the health centers, lack of information about ANC, service quality, age at first birth, educational background, place of living, sociocultural, and individual factors directly affected the utilization of antenatal care follow-up services (15–19). Adequate utilization of the recommended antenatal care visits is a key to protecting both the mother and newborn from the adverse complications of pregnancy and childbirth. Despite this fact, there is a paucity of evidence on ANC dropout and associated factors at the national level in Ethiopia. Meanwhile, this study was conducted to find out about the magnitude of ANC dropout and its factors among pregnant women in Ethiopia using the 2019 Ethiopia Mini Demographic and Health Survey (2019 EMDHS) data.

## Methods

### Study Design and Setting

In the 2019 Ethiopia Mini Demographic and Health Survey (2019 EMDHS), a community-based cross-sectional study was carried out from 21 March 2019 to 28 June 2019. Nine regional states [Afar, Tigray, Amhara, Oromia, Somali, Southern Nations, Nationalities, and Peoples' Region (SNNPR), Benishangul Gumuz, Gambella, and Harari] and two city administrations (Addis Ababa and Dire Dawa) are found in the country.

### Data Source

The Ethiopia Mini Demographic and Health Survey (EMDHS) data were used as a secondary data source for this study. A stratified two-stage cluster sampling was taken as the data source. Randomly, the enumeration areas (EAs) were selected in the first stage and then households were selected in the second stage. The target population for this study was all women who had any antenatal care in Ethiopia and those women in the selected enumeration areas (EAs) were the study population. Accordingly, a total weighted sample of 2,143 women who had antenatal care follow-up during their pregnancy was taken for this study.

### Study Variables

In this study, antenatal care (ANC) dropout was taken as the outcome variable. Those women who did not complete the recommended visits during their pregnancy (a minimum of four visits for normal pregnancy) were considered “Yes” for ANC dropout unless it was coded as “No” for the outcome variable. Age, religion, marital status, educational level, place of residence, region, wealth index, sex of head of household, age of head of household, the timing of 1st antenatal check, and getting laboratory investigation were considered expiratory variables in this study.

### Data Processing and Statistical Analysis

After data were extracted, editing, coding, and cleaning were done using Stata software version 16.0. The data were weighted using sampling weight (women's sample weight), primary sampling unit, and strata. Descriptive statistics and summary statistics were shown in the form of text, figures, and tables. Besides, the proportion of ANC dropout was presented using a pie chart.

Since the data had hierarchical and clustering nature, the mixed effect logistic model (multilevel model) was fitted to identify factors associated with ANC dropout. Due to the fact that the rate of ANC dropout varies from cluster to cluster, a cluster-level random intercept was introduced in the mixed effect logistic model. The within-cluster correlation was measured using the intracluster correlation coefficient (ICC), which is expected to be ≥10% to use the model. But, the ICC value was 7.77, which told us to select a fixed model (binary logistic regression model) over the mixed model. Variables having a *p*-value < 0.05 were considered as having a significant association with the ANC dropout. Model adequacy was also checked using Hosmer–Lemeshow test, which is expected to be ≥ 0.05 to say that the model is adequate.

## Results

### Characteristics of the Study Participants

The mean age of women who attended ANC follow-up in Ethiopia was 28.21 (±6.31 SD) and the average number of living children was 3.00 (±2.06). The majority of the mothers (91%) were married and 44.12% of them were orthodox. Nearly two-thirds (65.98%) of the respondents were from the rural areas. Eighty-five percentage of heads of the households were male and their mean age was 37.54 years (±12.6 SD). Nearly one-third (29.44%) of the mothers were from poor wealth index and 40.1% of the mothers were uneducated. Regarding the ANC follow-up, 50.05 and 7.56% of the respondents got their first visit in the second and third trimesters, respectively. Moreover, 96.38% of the mothers were sent to the laboratory during the ANC visits (Table 1).

**Table 1:** Background characteristics of women who had antenatal care follow-up in Ethiopia in 2021.

**Variables**	**Weighted frequency**	**Percent**
**Age**		
15–19	120	5.61
20–24	479	22.37
25–29	678	31.66
30–34	444	20.72
35–39	288	13.42
40–44	108	5.02
45–49	26	1.19
**Religion**		
Orthodox	946	44.12
Muslim	656	30.62
Protestant	527	24.60
Other^@^	14	0.66
**Marital status**		
Never married	24	1.10
Currently married	1,990	92.86
Formerly/ever married	129	6.04
**Educational level**		
Uneducated	842	39.29
Primary	886	41.35
Secondary	277	12.94
Higher	138	6.42
**Place of residence**		
Urban	729	34.02
Rural	1,414	65.98
**Region**		
Tigray	213	9.93
Afar	28	1.31
Amhara	555	25.91
Oromia	695	32.43
Somali	58	2.73
Benishangul	26	1.23
SNNPR	415	19.35
Gambella	15	0.70
Harari	8	0.39
Addis Ababa	115	5.36
Dire Dawa	14	0.66
**Wealth index**		
Poorest	253	11.80
Poorer	378	17.64
Middle	409	19.09
Richer	426	19.87
Richest	677	31.59
**Sex of head of household**		
Male	1,824	85.12
Female	319	14.88
**Age of head of household**		
15–19	9	0.42
20–24	98	4.58
25–29	400	18.68
30–34	418	19.51
35–39	422	19.68
40–44	316	14.75
45–49	176	8.20
>49	304	14.19
**Timing of 1st antenatal check**		
First trimester	908	42.39
Second trimester	1,073	50.06
Third trimester	162	7.56
**Got laboratory investigation**		
No	78	3.62
Yes	2,065	96.38

^@^*Catholic and traditional*.

### Magnitude of Antenatal Care Dropout

**Figure 1:**
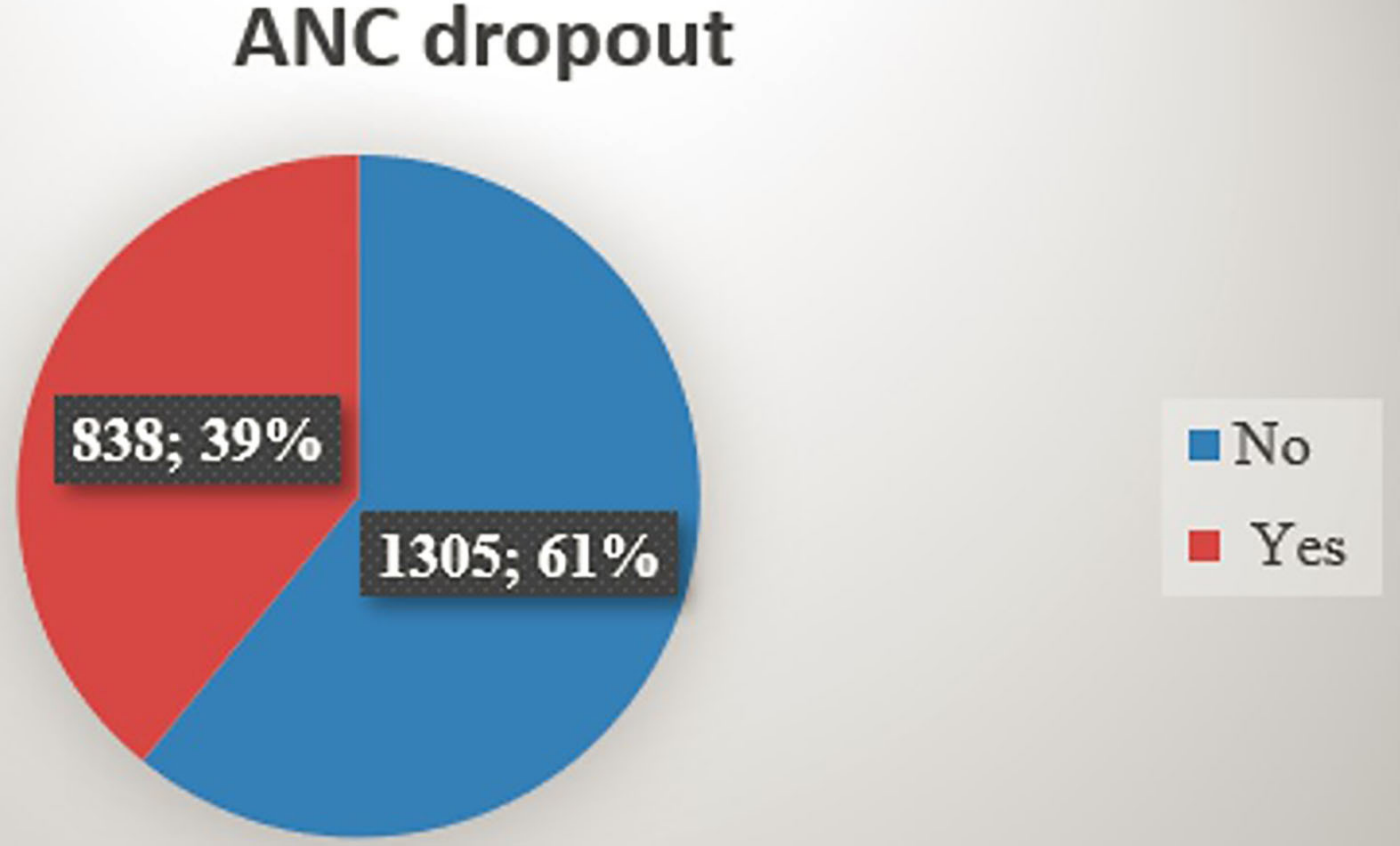
The magnitude of ANC dropout in Ethiopia, 2021.

The magnitude of ANC dropout was 39.12% (95% CI: 37.07 and 41.20%) among women who had antenatal care follow-up in Ethiopia (Figure 1).

### Factors Associated With Antenatal Care Dropout Among Pregnant Mothers in Ethiopia

In the multivariate logistic regression model, age, educational status, place of residence, the region where the mothers lived, gestational age at the time of 1st antenatal care visit, and access to laboratory service were found to be the significant associated factors of ANC dropout after controlling the confounder effect (Table 2).

**Table 2:** The bivariate and multivariate logistic regression analysis of ANC dropout among pregnant women in Ethiopia in 2021.

**Variables**	**ANC dropout**		**Odds Ratio (95% CI)**		***P*-value**
	**Yes**	**No**	**COR**	**AOR**	
**Age in years**					
15–29	523	755	1.00	1.00	
30–49	315	550	0.86 (0.73, 1.02)	0.71 (0.54, 0.94)	0.015*
**Religion**					
Orthodox	329	617	1.00	1.00	
Muslim	265	391	1.67 (1.38, 2.03)	1.17 (0.87, 1.56)	0.303
Protestant	238	289	2.40 (1.90, 3.04)	1.39 (0.97, 2.00)	0.075
Other^@^	7	7	7.25 (3.09, 17.03)	2.85 (1.08, 7.47)	0.034*
**Marital status**					
Currently married	760	1,230	1.00	1.00	1.00
Never married	10	14	1.28 (0.67, 2.43)	1.43 (0.65, 3.14)	0.374
Formerly married	61	68	1.20 (0.87, 1.64)	1.04 (0.69, 1.58)	0.835
**Educational level**					
Uneducated	385	457	1.00	1.00	1.00
Primary	361	525	0.74 (0.62, 0.90)	0.79 (0.62, 0.99)	0.050*
Secondary	68	209	0.49 (0.37, 0.64)	0.63 (0.44, 0.87)	0.008*
Higher	24	113	0.25 (0.17, 0.36)	0.39 (0.25, 0.62)	<0.001*
**Place of residence**					
Urban	202	527	1.00	1.00	
Rural	636	778	2.64 (2.19, 3.17)	1.53 (1.11, 2.10)	0.009*
**Region**					
Oromia	271	423	1.00	1.00	
Tigray	64	149	0.72 (0.50, 1.06)	1.12 (0.70, 1.80)	0.627
Afar	14	14	1.62 (1.10, 2.39)	1.86 (1.13, 3.06)	0.015*
Amhara	200	355	0.83 (0.57, 1.20)	1.08 (0.69, 1.71)	0.734
Somali	35	23	2.72 (1.60, 4.64)	3.24 (1.70, 6.18)	<0.001*
Benishangul	8	19	0.81 (0.54, 1.21)	0.98 (0.62, 1.54)	0.915
SNNPR	210	204	1.77 (1.22, 2.57)	1.54 (1.01, 2.36)	0.045*
Gambella	10	5	0.31 (0.51, 3.35)	3.92 (2.50, 6.13)	<0.001*
Harari	4	4	1.26 (0.87, 1.82)	3.28 (2.08, 5.17)	<0.001*
Addis Ababa	18	96	0.29 (0.18, 0.45)	0.94 (0.54, 1.63)	0.823
Dire Dawa	3	11	0.42 (0.27, 0.66)	0.90 (0.53, 1.52)	0.696
**Wealth index**					
Poorest	147	106	1.00	1.00	
Poorer	174	204	0.70 (0.53, 0.93)	0.90 (0.64, 1.26)	0.543
Middle	197	212	0.63 (0.47, 0.84)	0.97 (0.68, 1.38)	0.870
Richer	165	260	0.54 (0.40, 0.72)	0.86 (0.61, 1.23)	0.397
Richest	154	523	0.24 (0.19, 0.31)	0.74 (0.49, 1.11)	0.149
**Sex of head of household**					
Male	711	1,113	1.00	1.00	
Female	127	191	1.01 (0.83, 1.24)	0.90 (0.68, 1.19)	0.471
**Age of head of household**					
15–19	1	7	1.00	1.00	
20–24	43	56	1.85 (0.60, 5.66)	2.69 (0.81, 8.96)	0.106
25–29	155	245	1.47 (0.50, 4.29)	2.13 (0.67, 6.78)	0.203
30–34	154	264	1.67 (0.57, 4.87)	2.36 (0.73, 7.61)	0.150
35–39	164	257	1.61 (0.55, 4.71)	2.53 (0.78, 8.23)	0.124
40–44	126	190	1.41 (0.48, 4.16)	2.12 (0.64, 7.03)	0.220
45–49	72	103	1.72 (0.57, 5.17)	2.44 (0.72, 8.28)	0.152
50–54	39	71	1.51 (0.49, 4.59)	2.19 (0.63, 7.56)	0.217
55–59	13	24	1.47 (0.45, 4.81)	2.47 (0.66, 9.17)	0.178
>59	70	88	1.78 (0.59, 5.36)	2.51 (0.75, 8.46)	0.137
**Timing of 1st antenatal check**					
Second trimester	478	596	1.00	1.00	
First trimester	206	703	0.41 (0.34, 0.49)	0.49 (0.40, 0.60)	<0.001*
Third trimester	156	6	24.9 (10.9, 56.9)	23.7 (10.78, 55.1)	<0.001*
**Got laboratory investigation during 1st antenatal check**					
No	54	24	1.00	1.00	
Yes	784	1,281	0.16 (0.09, 0.30)	0.25 (0.13, 0.51)	<0.001*
**Number of living children (mean** **±SD)**	3.24 (±2.16)	2.89 (±1.99)	1.16 (1.10, 1.23)	1.00 (0.94, 1.06)	0.989

*P-value < 0.05, AOR, adjusted odds ratio; COR, crud odds ratio; SD, standard deviation. ^@^Catholic and traditional.

The odds of ANC dropout decreased by 21% [adjusted odds ratio (AOR) = 0.79 (0.62, 0.99)], 37% [AOR = 0.63 (9.44, 0.87)], and 61% [AOR = 0.39 (0.25, 0.62)] among pregnant mothers who were on ANC follow-up and who had primary, secondary, and higher education, respectively, as compared to pregnant mothers with no formal education. The likelihood of ANC dropout was 1.53 times [AOR = 1.53 (1.11, 2.10)] higher among the pregnant mothers who lived in rural areas as compared with the pregnant mothers who lived in urban areas.

The odds of ANC dropout were 1.86 [AOR = 1.86 (1.13, 3.06)], 3.24 [AOR = 3.24 (1.70, 6.18)], 1.54 [AOR = 1.54 (1.01, 2.36)], 3.92 [AOR = 3.92 (2.50, 6.13)], and 3.28 times [AOR = 3.28 (2.08, 5.17)] higher in Afar, Somali, SNNPR, Gambella, and Harari among pregnant mothers as compared to the mothers who lived in Oromia at the time of the survey. The likelihoods of defaulting the ANC services were 39% [AOR = 0.71; 95% CI: (0.54, 0.94)] lower among the pregnant mothers aged between 30 and 49 years as compared with the pregnant mothers aged between 15 and 29 years.

The gestational age at the time of the visit was another significant associated factor for ANC dropout. The odds of ANC dropout decreased by 51% [AOR = 0.49 (0.40, 0.60)] for the mothers who started ANC visit in the first trimester of their pregnancy as compared with the mothers who started ANC visit in the second trimester of their pregnancy. However, the odds of ANC dropout were 23.7 times [AOR = 23.7 (10.78, 55.1)] higher for the pregnant mothers who had their first ANC visit in the third trimester as compared with the pregnant mothers who had their first ANC visit in the second trimester.

The likelihood of defaulting from ANC service decreased by 75% [AOR = 0.25 (0.13, 0.51)] for the mothers who have received laboratory service at the time of the survey as compared with the mothers who did not receive laboratory service at the time of the survey.

## Discussion

This study assessed the magnitude and associated factors of ANC dropout using the 2019 EMDHS data. Accordingly, the magnitude of ANC dropout among women on ANC follow-up in Ethiopia was found to be 39.12% (95% CI: 37.07 and 41.20%). The finding is higher than previous studies conducted in Debre Markos (32.2%) (18) and Nigeria (16). This might be due to the difference in accessibility and quality of maternal health services. The Debre Markos study was conducted among urban women in Debre Markos where the accessibility and quality of maternal health services are better, whereas the current study was conducted among the general population of women in Ethiopia. Regarding the study in Nigeria, women tend to have higher access to maternity care and lower dropout due to higher socioeconomic status. This implies that though Ethiopian pregnant mothers have access to health institutions, there is a significant gap in keeping the continuum of maternal care.

The current study also identified factors associated with ANC dropout. Pregnant mothers who were on ANC follow-up and who had primary, secondary, and higher education were less likely to drop out from recommended ANC visits compared to those with no formal education. This finding is in line with previous studies carried out in the Enemay district of Ethiopia (20), Pakistan (17), and Egypt (21). This might be due to the positive association between women's education and ANC service. Those with better knowledge of the importance and adequacy of ANC services are more likely to utilize the recommended number of ANC visits (22). This might imply that educating women will increase the utilization of ANC services.

The likelihood of ANC dropout is higher among pregnant mothers from rural area. This finding is in agreement with previous studies in Nigeria (23, 24) and Indonesia (25) and this might be due to the low accessibility of ANC service in rural areas. The other reason might be being far away from health facilities and multiple responsibilities among most women in rural area that hinder them to get enough time for seeking the service.

Pregnant women in Afar, Somali, SNNPR, Gambella, and Harari regions had higher odds of ANC dropout compared to the women who lived in Oromia regional state. The reason for this discrepancy could be the difference in accessibility and quality of service across different geographical regions of Ethiopia. This could be evidenced by a higher number of health facilities in the region compared to other regions of Ethiopia (26).

The age of women was another variable significantly associated with ANC dropout. Pregnant women of higher age were at lower odds of ANC dropout. This finding is similar to the result from a previous study (27) and this might be explained by the fact that women of higher age are more likely to have the autonomy in healthcare decision-making that enables them to decide for receiving the care by themselves, which, in turn, increases the chance of pregnant women to use ANC services. This implies that early age at marriage also adversely affects ANC utilization.

This study revealed that the odds of ANC dropout is lower for the mothers who started ANC visit early in the first trimester of their pregnancy. This might be justified by early ANC visit that provides an opportunity for women to get information on the importance of ANC services and subsequent schedules and this could motivate and encourage them to have the recommended number of ANC visit (28, 29).

The likelihood of defaulting from ANC service was lower for the women who received laboratory service at the time of the survey as compared to those mothers who did not receive the laboratory service at the time of the survey. This result is in agreement with previous studies in Senegal (30) and this might be explained by the fact that those women who received laboratory service had a chance to get professional advice based on laboratory findings and this could increase women's understanding of the importance of ANC and their health condition, which, in turn, encourages women to complete their ANC follow-up.

The main limitation of the current study was that it was conducted based on secondary data; thus, we could not include information on some of the important variables such as the quality of ANC services and women's decision-making autonomy among others. The lack of sufficient literature on the topic was among the additional limitations faced for comparison of our findings with previous studies in the discussion part.

## Conclusion

More than one-third of the pregnant women in Ethiopia have dropped out from their ANC follow-up in the study period. Being old-aged, educated and urban residents, having the first ANC visit in the first trimester, and having access to laboratory services were positively associated with ANC dropouts. Therefore, we recommended encouraging women to have ANC visit at an early stage of pregnancy and conducting basic laboratory investigations during their visit. When undertaking that, due attention should be given to the young, uneducated, and rural dweller women, particularly, in Afar, Somali, SNNPR, Gambella, and Harari regions.

## Data Availability Statement

The original contributions presented in the study are included in the article/supplementary material, further inquiries can be directed to the corresponding author/s.

## Author Contributions

The conception of the work, design of the work, acquisition of data, analysis, and interpretation of data were conducted by BT. Data curation, drafting the article, revising it critically for intellectual content, validation, and final approval of the version to be published were done by MW, BT, AW, NB, and DE. All authors have read and approved the final version of the manuscript.

## Conflict of Interest

The authors declare that the research was conducted in the absence of any commercial or financial relationships that could be construed as a potential conflict of interest.

## Publisher's Note

All claims expressed in this article are solely those of the authors and do not necessarily represent those of their affiliated organizations, or those of the publisher, the editors and the reviewers. Any product that may be evaluated in this article, or claim that may be made by its manufacturer, is not guaranteed or endorsed by the publisher.
